# Association of dietary long-chain *n*-3 fatty acid intake with depression severity in USA adults: a population-based cross-sectional study

**DOI:** 10.1017/S0007114526106424

**Published:** 2026-06-14

**Authors:** Joseph Vinod Varre, Guadalupe Márquez-Velarde, Mario Suárez, Heidi J. Wengreen, Mia Dustin, Stephan van Vliet

**Affiliations:** 1 Nutrition, Dietetics, and Food Sciences, Utah State University, USA; 2 Department of Sociology, Social Work, and Anthropology, Utah State University, USA; 3 School of Teacher Education and Leadership, Utah State University, USA

**Keywords:** *n*-3 fatty acids, Depression, Patient Health Questionnaire-9: National Health and Nutrition Examination Survey, Dietary intake, Cross-sectional study

## Abstract

Depression affects over 280 million people worldwide. Long-chain *n*-3 fatty acids may relate to depression, but observational evidence is inconsistent. This cross-sectional analysis of the National Health and Nutrition Examination Survey 2021–2023 examined the association between dietary long-chain *n*-3 intake and depression severity in USA adults ≥ 18 years with complete dietary, Patient Health Questionnaire-9 (PHQ-9) and covariate data (*n* 3608). PHQ-9 severity categories (0–4 to 20–27) served as the main outcome. Total *n*-3 (α-linolenic acid (ALA), EPA, DPA and DHA) from 24-h recalls (Food and Nutrient Database for Dietary Studies 2021–2023) served as the exposure; supplements were excluded, and supplement use was a binary covariate. Survey-weighted ordinal logistic regression (svyolr) was used with all continuous variables centred/scaled (OR per 1 sd). Covariates included age, sex, race/ethnicity (collapsed for sparse cells), income:poverty ratio, BMI, smoking, alcohol, physical activity and *n*-3 supplement use. Higher total *n*-3 intake was inversely associated with depression severity (OR 0·865 per 1 sd, 95 % CI 0·761, 0·983, *P* = 0·026). EPA showed a significant inverse association (OR 0·907, 95 % CI 0·824, 0·998, *P* = 0·045); ALA, DPA and DHA were NS. No interaction by sex (*P* = 0·656) or race/ethnicity (*P* = 0·155). Sensitivity analyses: excluding supplement users (*n* 3093) OR 0·872 (95 % CI 0·773, 0·984, *P* = 0·026); two recalls only (*n* 3229) OR 0·847 (95 % CI 0·751, 0·955, *P* = 0·007). Dietary *n*-3 intake, particularly EPA, was modestly and inversely associated with depression severity. Residual confounding and reverse causation remain possible; longitudinal studies with biomarkers are needed.

Depression is a leading cause of disability worldwide, affecting approximately 280 million people^(1)^. In the USA, the prevalence of major depressive disorder among adults is 8·4 %^(2)^. Although pharmacological and psychological interventions exist, treatment resistance remains common, highlighting the need for preventive strategies^(3)^. In this context, dietary patterns have emerged as modifiable factors associated with mental health outcomes, with a systematic review and meta-analysis of community-dwelling adults reporting lower odds of depression among individuals adhering to healthier dietary patterns and higher odds among those consuming Western-style diets^(4)^. *n*-3 fatty acids, particularly EPA and DHA, are incorporated into neuronal membranes and influence their fluidity and function. They serve as precursors for anti-inflammatory eicosanoids and specialised pro-resolving mediators, potentially reducing neuroinflammation implicated in depression. *n*-3 fatty acids also modulate neurotransmitter systems (serotonin and dopamine), affect neuroplasticity and neurogenesis and may regulate the hypothalamic–pituitary–adrenal axis while reducing oxidative stress. Beyond fatty acids, other bioactive components of whole dietary patterns, such as polyphenols commonly found in Mediterranean-style diets, have also been associated with improvements in depressive symptoms, potentially through antioxidant, anti-inflammatory and gut–brain axis–related mechanisms^(5)^. In contrast, a high *n*-6 intake relative to *n*-3 intake may promote pro-inflammatory processes. Emerging evidence suggests that long-chain *n*-PUFA, primarily EPA and DHA from marine sources, may play a role in mental health. Meta-analyses of randomised controlled trials have shown mixed results regarding *n*-3 supplementation for depression, with some suggesting modest benefits for EPA-predominant formulations^(6,7)^. However, evidence from large-scale observational studies examining habitual dietary *n*-3 intake in the general population is limited and inconsistent^(8,9)^. National Health and Nutrition Examination Survey (NHANES) 2021–2023 provides a unique opportunity to examine this relationship in a nationally representative sample using standardised assessment methods, with detailed dietary recall data from which individual *n*-3 fatty acid intakes can be derived and validated depression screening using the Patient Health Questionnaire-9 (PHQ-9)^(10)^. This study aimed to examine the cross-sectional association between dietary intake of total and individual long-chain *n*-3 fatty acids (alpha-linolenic acid (ALA), EPA, DPA and DHA) and depression severity in USA adults.

## Methods

### Study population

**Figure 1.**
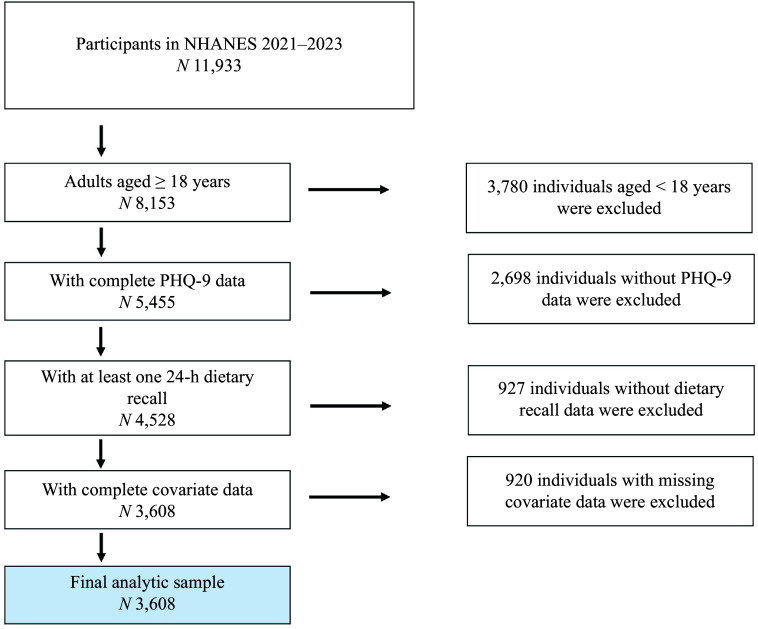
Flow chart of participant selection. Flow chart of participant selection from the NHANES 2021–2023. The final analytic sample consisted of 3608 adults aged ≥ 18 years with complete data on depression severity (PHQ-9), dietary *n*-3 intake and all covariates. NHANES, National Health and Nutrition Examination Survey; PHQ-9, Patient Health Questionnaire-9.

Data were obtained from the NHANES 2021–2023, a continuous, nationally representative, cross–sectional survey using a complex, multistage probability sampling design to select participants representative of the civilian, non-institutionalised USA population. The NHANES protocol was approved by the National Center for Health Statistics Research Ethics Review Board, and all participants provided informed consent in writing. This secondary analysis of de-identified, publicly available data were classified as exempt by the Utah State University Institutional Review Board (protocol #14827). We included adults aged ≥ 18 years who completed the depression questionnaire, had at least one 24-h dietary recall and had complete data on all covariates. The final analytical sample comprised 3608 participants (Figure 1).

### Depression assessment

Depression severity was assessed using the Patient Health Questionnaire-9 (PHQ-9), a validated self-administered tool based on nine Diagnostic and Statistical Manual of Mental Disorders, 5th Edition criteria for major depressive disorder^(11)^. Participants rated the frequency of depressive symptoms over the past 2 weeks on a four-point scale (0 = not at all to 3 = nearly every day). Total scores (0–27) were categorised as follows: None (0–4), Mild (5–9), Moderate (10–14), Moderately Severe (15–19) and Severe (20–27).

### Dietary assessment and fatty acid derivation

Dietary intake was assessed using two 24-h dietary recalls conducted by trained interviewers using the United States Department of Agriculture of Agriculture Automated Multiple-Pass Method. The first recall was in-person during the Mobile Examination Center visit, and the second was by telephone 3–10 d later. Individual fatty acid intakes (*α*-linolenic acid (ALA, 18:3*n*-3), EPA, 20:5*n*-3, DPA, 22:5*n*-3 and DHA, 22:6*n*-3) were derived from dietary recall data using the United States Department of Agriculture Food and Nutrient Database for Dietary Studies 2021–2023^(12)^. The Food and Nutrient Database for Dietary Studies provides nutrient composition values for all foods reported in the dietary recall. Fatty acid intakes were calculated as follows: for participants with both Day 1 and Day 2 recalls, average intake across both days; for participants with only Day 1 recall, Day 1 intake only. The total *n*-3 fatty acid intake was calculated as the sum of ALA, EPA, DPA and DHA, expressed in grams per day.

Supplement Use: *n*-3 supplement use was identified using the Dietary Supplement Questionnaire by text matching of supplement names. Supplements containing ‘fish oil,’ ‘*n*,’ ‘EPA,’ ‘DHA,’ ‘flax’ or ‘krill’ were classified as *n*-3 fatty acid supplements. The dietary fatty acid intakes reported in this analysis exclude supplement sources and represent dietary intake only. *n*-3 supplement use was included as a binary covariate (yes/no) in all the models.

### Covariates

Covariates were selected a priori based on potential associations with both *n*-3 intake and depression.

Demographics: Age (continuous, years), sex (male/female), race/ethnicity (Mexican American, other Hispanic, non-Hispanic White (reference) and other). Due to sparse cell counts in some race/ethnicity categories, non-Hispanic Black, non-Hispanic Asian and Other/Multiracial were collapsed into a single ‘Other’ category for analysis.

Socio-economic: Ratio of family income to poverty threshold (continuous)

Lifestyle: BMI (continuous, kg/m^2^), ever smoked ≥ 100 cigarettes in lifetime (yes/no), alcohol consumption (days per year, continuous) and physical activity (minutes of moderate activity per week, continuous)

Medication: *n*-3 supplement use (yes/no)

All covariates were assessed using standardised NHANES questionnaires and examination protocols. BMI was calculated based on the measured height and weight.

Antidepressant Medication Use: NHANES 2021–2023 prescription medication files do not allow reliable classification of antidepressant use relative to PHQ-9 symptom timing. We were unable to identify or control for antidepressant use, which is an important unmeasured confounder. This limitation is discussed in detail in the limitations section.

### Statistical analysis

All analyses accounted for the complex sampling design of the NHANES, incorporating sample weights, strata and primary sampling units. A survey design object was created using the ‘svydesign’ function in R, with ‘nest = TRUE’ to account for the nested structure of primary sampling units within strata. The ‘options (survey.lonely.psu = ‘adjust’)’ setting was applied to handle the strata with single primary sampling units. Dietary Day 1 weights (WTDRD1) were applied for analyses, including participants with a single recall, consistent with the NHANES analytic guidance^(13)^. Participants with two dietary recalls contributed average intake values but were analysed using Day 1 dietary weights, consistent with the NHANES guidance for mixed recall availability. Descriptive statistics were calculated for the total sample and stratified by depression severity. Continuous variables are presented as weighted means (standard deviations), and categorical variables are presented as weighted frequencies (percentages). The association between *n*-3 fatty acid intake and depression severity was examined using survey-weighted ordinal logistic regression (proportional odds model) via the ‘svyolr’ function, treating depression severity as an ordered categorical variable (0 = None to 4 = Severe). The proportional-odds assumption was assessed qualitatively. Because standard Brant testing is not directly applicable to the survey-weighted ordinal model, we examined whether the cumulative-logit relationships appeared approximately parallel across outcome thresholds; no major departures were evident.

Variable Scaling: All continuous variables (age, BMI, income:poverty ratio, alcohol consumption, physical activity and *n*-3 fatty acid intake) were centred and scaled to a mean of 0 and a standard deviation of 1 prior to model fitting. This scaling facilitates the interpretation of odds ratios per 1 sd) increase, improves model convergence and addresses potential multicollinearity issues.

Three models were specified.

Model 1: Unadjusted

Model 2: Adjusted for age, sex and race/ethnicity

Model 3: Fully adjusted (age, sex, race/ethnicity, income:poverty ratio, BMI, smoking status, alcohol use, physical activity and *n*-3 supplement use)

Model 3 represents the primary analyses. Results are presented as OR with 95 % CI and *P* values. All OR are expressed as per a 1 sd increase in the exposure variable. OR < 1·0 indicate an inverse association (higher *n*-3 intake associated with lower odds of high depression severity). Separate models were run for total *n*-3 intake and for each individual fatty acid (ALA, EPA, DPA and DHA). Interaction Analyses: Potential effect modification by sex and race/ethnicity was assessed by including multiplicative interaction terms (*n*-3 × sex, *n*-3 × race/ethnicity) in the fully adjusted models. Sensitivity Analyses: We conducted analyses (1) excluding *n*-3 supplement users and (2) restricting participants to those with two complete dietary recalls. Missing Data: Complete case analysis was performed. Participants with missing data on any of the variables were excluded. All analyses were conducted using R version 4.5.0 with the ‘survey’ package for conducting survey-weighted analyses. Statistical significance was set at *P* < 0·05 (two-tailed).

## Results

### Sample characteristics

The analytical sample included 3608 adults aged ≥ 18 years. Table 1 presents the weighted participant characteristics by depression severity category. Participants with no depression (PHQ-9 score 0–4) comprised 67·6 % of the sample (*n* 2438), followed by mild depression (20·5 %, *n* 739), moderate depression (7·7 %, *n* 279), moderately severe depression (2·8 %, *n* 101) and severe depression (1·4 %, *n* 51). The mean age decreased with increasing depression severity, from 55·8 years in the no depression category to 45·1 years in the severe depression category. The proportion of female participants was higher in the moderate and moderately severe depression categories (65·9 % and 63·4 %, respectively) than in the no depression category (51·8 %). The mean BMI increased with depression severity, ranging from 29·2 kg/m² in the no depression category to 32·6 kg/m² in the moderately severe category. *n*-3 supplement use was reported by 15·4 % of participants in the no depression category and decreased to 9·8 % in the severe depression category.

**Table 1.** Participant characteristics by depression severity category (*n* 3608) Table 1 long description.

Depression severity (PHQ-9)	*n*	%	Age, years		Female		BMI, kg/m²		Total *n*-3, g/d		ALA, g/d		EPA, g/d		DHA, g/d		*n*-3 supplement use	
			Mean	sd	*n*	%	Mean	sd	Mean	sd	Mean	sd	Mean	sd	Mean	sd	*n*	%
None (0–4)	2438	67·6	55·8	16·4	1263	51·8	29·2	6·7	2·09	1·34	1·90	1·22	0·061	0·172	0·085	0·234	375	15·4
Mild (5–9)	739	20·5	50·2	18·2	454	61·4	30·6	8·0	1·95	1·39	1·80	1·32	0·050	0·173	0·066	0·232	98	13·3
Moderate (10–14)	279	7·7	47·6	17·8	184	65·9	31·5	8·8	1·80	1·13	1·69	1·04	0·035	0·109	0·049	0·158	27	9·7
Moderately severe (15–19)	101	2·8	50·1	17·9	64	63·4	32·6	9·2	1·85	1·29	1·79	1·39	0·017	0·049	0·020	0·069	10	9·9
Severe (20–27)	51	1·4	45·1	16·7	29	56·9	30·4	8·4	1·59	1·26	1·50	1·22	0·029	0·137	0·036	0·184	5	9·8

ALA, *α*-linolenic acid.

Values were weighted to represent the USA civilian, non-institutionalised population. *n* represents the unweighted sample size; percentages and means are weighted.

### Association between *n*-3 intake and depression severity

Table 2 presents the results of the survey-weighted ordinal logistic regression models examining the association between total *n*-3 intake and depression severity. Model 1 (unadjusted) showed convergence issues and is not reported. Model 2 (adjusted for age, sex and race/ethnicity) also showed convergence issues and is not reported. In the fully adjusted model (Model 3), which included age, sex, race/ethnicity, income:poverty ratio, BMI, smoking status, alcohol use, physical activity and *n*-3 supplement use, higher total *n*-3 intake was inversely associated with depression severity (OR 0·865 per 1 sd increase, 95 % CI 0·761, 0·983, *P* = 0·026).

**Table 2.** Association of total *n*-3 fatty acid intake with depression severity: survey-weighted ordinal logistic regression models Table 2 long description.

Model	Adjustment	OR per 1 sd increase	95 % CI	*P*
Model 1	Unadjusted	–	–	–
Model 2	Age, sex, race/ethnicity	–	–	–
Model 3	Fully adjusted^*^	0·865	0·761, 0·983	0·026

* The fully adjusted model included age, sex, race/ethnicity, income:poverty ratio, BMI, smoking status, alcohol use, physical activity and *n*-3 supplement use. All continuous variables were scaled and centred. The OR represent the odds of higher depression severity per one sd increase in dietary *n*-3 intake. Models 1 and 2 are not reported due to convergence issues.

### Individual fatty acids

Table 3 presents the results for individual *n*-3 fatty acids in fully adjusted models. EPA showed a statistically significant inverse association with depression severity (OR 0·907 per 1 sd increase, 95 % CI 0·824, 0·998, *P* = 0·045). ALA showed a non-significant inverse association (OR 0·918 per 1 sd increase, 95 % CI 0·767, 1·098, *P* = 0·348). DPA showed a non-significant inverse association (OR 0·911 per 1 sd increase; 95 % CI 0·819, 1·012, *P* = 0·083). DHA showed a non-significant inverse association (OR 0·919 per 1 sd increase, 95 % CI 0·841, 1·004, *P* = 0·061).

**Table 3.** Association of individual *n*-3 fatty acids with depression severity: fully adjusted survey-weighted models Table 3 long description.

Fatty acid	Unit	OR	95 % CI	*P*
ALA (18:3*n*-3)	Per 1 sd increase	0·918	0·767, 1·098	0·348
EPA (20:5*n*-3)	Per 1 sd increase	0·907	0·824, 0·998	0·045
DPA (22:5*n*-3)	Per 1 sd increase	0·911	0·819, 1·012	0·083
DHA (22:6*n*-3)	Per 1 sd increase	0·919	0·841, 1·004	0·061

ALA, *α*-linolenic acid.

All models were fully adjusted for age, sex, race/ethnicity, income:poverty ratio, BMI, smoking status, alcohol use, physical activity and *n*-3 supplement use. All continuous variables were scaled and centred. OR represent the odds of higher depression severity per one sd increase in fatty acid intakes.

### Interaction analyses

The association between total *n*-3 intake and depression severity did not differ significantly by sex (*n*-3 × sex interaction, *P* = 0·656) or race/ethnicity (*n*-3 × race/ethnicity interaction, *P* = 0·155).

### Sensitivity analyses

Table 4 presents the sensitivity analysis results. After excluding *n*-3 supplement users (*n* 3093), the inverse association remained statistically significant (OR 0·872 per 1 sd increase, 95 % CI 0·773, 0·984, *P* = 0·026). Restricting the analysis to participants with two complete dietary recalls (*n* 3229) yielded a stronger inverse association (OR 0·847 per 1 sd increase, 95 % CI 0·751, 0·955, *P* = 0·007).

**Table 4.** Sensitivity analyses: association of total *n*-3 intake with depression severity Table 4 long description.

Analysis	*n*	OR per 1 sd increase	95 % CI	*P*
Main analysis (reference)	3608	0·865	0·761, 0·983	0·026
Excluding *n*-3 supplement users	3093	0·872	0·773, 0·984	0·026
Two dietary recalls only	3229	0·847	0·751, 0·955	0·007

All models were fully adjusted for age, sex, race/ethnicity, income:poverty ratio, BMI, smoking status, alcohol use, physical activity and *n*-3 supplement use (where applicable). All continuous variables were scaled and centred. OR represent the odds of higher depression severity per standard deviation increase in total *n*-3 intake.

## Discussion

In this nationally representative cross-sectional study of USA adults, dietary *n*-3 fatty acid intake was inversely associated with depression severity after comprehensive adjustment for demographic, socio-economic, lifestyle and health-related confounders. As shown in Table 2, the fully adjusted model (Model 3) demonstrated a statistically significant inverse association between total *n*-3 intake and depression severity (OR 0·865 per 1 sd increase, 95 % CI 0·761, 0·983, *P* = 0·026). Among the individual fatty acids, EPA showed a significant inverse association (OR 0·907 per 1 sd increase, 95 % CI 0·824, 0·998, *P* = 0·045), whereas ALA, DPA and DHA showed non-significant but consistent inverse associations (Table 3). There was no evidence of effect modification by sex (*P* = 0·656) or race/ethnicity (*P* = 0·155). The persistence of the inverse association after comprehensive covariate adjustment suggests that the relationship may be independent of measured demographic, socio-economic and lifestyle factors, although unmeasured confounding remains a possibility.

The magnitude of the observed association, although statistically significant, was modest in clinical terms. A one sd increase in total *n*-3 intake was associated with approximately 14 % lower odds of high depression severity (Table 2). This effect size suggests that dietary *n*-3 intake may contribute to population-level differences in depression severity rather than representing a primary intervention strategy for individual treatments. This modest magnitude is consistent with the multifactorial nature of depression, in which dietary factors likely operate alongside genetic, environmental and psychosocial determinants. At the population level, even modest associations can have meaningful public health implications if they reflect modifiable dietary patterns that are part of the broader lifestyle factors associated with mental health^(14)^. This association persisted after adjusting for multiple potential confounders, including age, sex, race/ethnicity, socio-economic status, BMI, smoking, alcohol use, physical activity and *n*-3 supplement use, suggesting a potential independence from these measured factors.

The finding that EPA showed a statistically significant inverse association (OR 0·907 per 1 sd increase, 95 % CI 0·824, 0·998, *P* = 0·045), whereas other individual fatty acids did not reach statistical significance (Table 3), warrants cautious interpretation. EPA is primarily derived from marine sources, particularly fatty fish, and its association may reflect broader dietary patterns rather than the specific biological effect of EPA^(15)^. Fish consumption is often associated with healthier dietary patterns, higher socio-economic status and lifestyle factors that may independently influence mental health^(16)^. The non-significant associations observed for ALA, DPA and DHA (Table 3) do not necessarily indicate that these fatty acids are less relevant; rather, they may reflect measurement challenges^(17)^, differential bioavailability^(18,19)^ or the possibility that EPA serves as a marker for a dietary pattern rich in marine *n*-3 sources^(20)^. The borderline non-significant association for DHA (OR 0·919, 95 % CI 0·841, 1·004, *P* = 0·061) suggests that marine *n*-3 fatty acids may be relevant; however, the current analysis cannot distinguish between direct effects and dietary pattern associations. This finding is consistent with previous research suggesting that EPA may be more strongly associated with depression outcomes than DHA, although the biological mechanisms underlying potential EPA-specific effects remain unclear and require further investigation^(21)^.

The consistency of the findings across the sensitivity analyses strengthens the confidence in the robustness of the observed associations. As shown in Table 4, excluding *n*-3 supplement users (*n* 3093) yielded a similar inverse association (OR 0·872 per 1 sd increase, 95 % CI 0·773, 0·984, *P* = 0·026), indicating that the association was not driven by supplement use patterns. Restricting the analysis to participants with two complete dietary recalls (*n* 3229) produced a stronger inverse association (OR 0·847 per 1 sd increase, 95 % CI 0·751, 0·955, *P* = 0·007), which may reflect a more accurate dietary assessment or the selection of participants with more stable dietary patterns. The persistence of statistically significant associations across these different analytical approaches, despite varying sample sizes and dietary assessment completeness, suggests that the observed relationship is not an artifact of measurement error or confounding factors such as supplement use. This consistency across sensitivity analyses, combined with the comprehensive covariate adjustment in the primary model, supports the robustness of the findings while acknowledging the limitations inherent in cross-sectional observational data.

These findings are consistent with those of previous observational studies reporting inverse associations between *n*-3 intake or status and depression^(22)^. However, meta-analyses of randomised controlled trials have shown limited evidence for *n*-3 supplementation in the prevention or treatment of depression in the general population^(23,24)^. The apparent discrepancy between observational studies reporting inverse associations and randomised controlled trials showing limited evidence for *n*-3 supplementation may reflect fundamental differences in exposure characteristics rather than contradictory evidence. Observational studies capture long-term habitual dietary intake patterns that develop over years, whereas randomised controlled trials typically examine short-term supplementation (often weeks to months) in participants who may already have adequate baseline *n*-3 status^(25,26)^. The dietary patterns associated with higher *n*-3 intake in observational studies, such as regular fish consumption as part of a Mediterranean-style diet, represent complex, sustained exposures that may differ substantially from isolated supplement interventions. Additionally, randomised controlled trials often enroll participants with clinical depression or specific depression subtypes, whereas population-based observational studies capture the full spectrum of depression severity^(27)^. The complementarity of these research approaches suggests that dietary *n*-3 intake may be more relevant for population-level mental health patterns and primary prevention contexts^(6,28)^, whereas supplementation trials may be better suited for examining therapeutic effects in specific clinical populations^(7,28)^. The consistency of our findings with those of other observational studies, combined with the limitations of short-term supplementation trials in addressing long-term dietary patterns, suggests that both research approaches contribute valuable but distinct insights into the relationship between *n*-3 fatty acids and mental health.

This study has notable strengths, including a large, nationally representative sample with standardised assessment methods; detailed *n*-3 fatty acid assessment using the United States Department of Agriculture Food and Nutrient Database for Dietary Studies; examination of individual fatty acids; comprehensive adjustment for confounders; use of survey-weighted analyses to account for complex sampling design and consistency across sensitivity analyses. However, several important limitations must be considered when interpreting these findings. First, the cross-sectional design of the study did not allow for the establishment of causality. The temporal relationship between *n*-3 intake and depression cannot be determined, and reverse causation remains possible (e.g., depression may influence dietary choices or appetite). Second, dietary intake was assessed using 24-h recalls, which reflect short term rather than habitual dietary consumption. Fatty acid intake varies substantially day-to-day, and two recalls may not adequately capture the long-term patterns. This is particularly relevant for *n*-3 fatty acids that are consumed episodically (e.g., fish). Measurement error in *n*-3 intake assessment would likely bias the results towards the null, although some error may remain. Future studies integrating dietary data with erythrocyte or plasma *n*-3 biomarkers may better distinguish true associations from exposure misclassification. Third, dietary fatty acid intake did not include supplementation. While we controlled for supplement use as a covariate, total *n*-3 exposure (dietary + supplements) may have been underestimated in our primary exposure variable. However, sensitivity analyses excluding supplement users yielded similar results, suggesting that this limitation did not substantially affect our findings. Fourth, we were unable to control for the use of antidepressant medications. The NHANES 2021–2023 prescription medication files do not allow for the reliable classification of antidepressant use relative to the PHQ-9 symptom timing. This represents a critical unmeasured confounder, as individuals taking antidepressants would be expected to have higher depression severity scores (if the medication is ineffective) or lower scores (if the medication is effective). The inability to stratify by treatment status or adjust for medication use substantially limited causal inference from these findings. Fifth, residual confounding is possible, despite adjusting for multiple confounders. Unmeasured factors such as overall diet quality, stress, social support and genetic factors could influence both *n*-3 intake and depression risk. Sixth, depression was assessed using the PHQ-9, a screening tool, rather than a clinical diagnosis. Whilst well-validated, it may not capture all depression aspects or distinguish subtypes that might respond differently to *n*-3 fatty acids. Finally, our findings may not generalise to populations with clinical depression, specific depression subtypes or individuals with very low *n*-3 status.

These findings suggest an inverse association between dietary *n*-3 intake, particularly EPA, and depression severity in USA adults. However, the cross-sectional design and potential for unmeasured confounding factors limit causal inference. The observed associations may reflect shared lifestyle or socio-economic factors rather than the direct biological effects of *n*-3 fatty acids. The persistence of associations after comprehensive adjustment suggests potential independence from measured confounders, although unmeasured confounding remains possible. These findings do not support population-level recommendations for *n*-3 supplementation for depression prevention, given the limited evidence from randomised controlled trials. However, these findings are consistent with dietary guidelines promoting fish consumption as part of a healthy dietary pattern. Future research should focus on longitudinal studies that establish temporal relationships and account for changes in *n*-3 intake over time. Studies incorporating objective biomarkers of *n*-3 status would address dietary assessment limitations and provide more accurate exposure characterisation. Research examining effect modification by depression subtype, *n*-3 status, genetic factors or inflammatory markers may help identify the subgroups most likely to benefit from *n*-3 interventions. Additionally, studies examining the role of dietary patterns rich in *n*-3 sources (e.g., Mediterranean-style diets) may provide insights into the broader context in which *n*-3 fatty acids influence mental health outcomes.

### Conclusion

In this nationally representative cross-sectional study, dietary long-chain *n*-3 fatty acid intake was inversely associated with depression severity after comprehensive covariate adjustment (OR 0·865 per 1 sd increase, 95 % CI 0·761, 0·983, *P* = 0·026). EPA showed a particularly strong inverse association (OR 0·907 per 1 sd increase, *P* = 0·045). However, the cross-sectional design and potential for unmeasured confounding, particularly by the use of antidepressant medication, limit causal inference. Longitudinal studies with biomarker measurements are needed to establish temporal relationships and further elucidate this relationship.

## Table 1 Long description

A table with 3608 participants categorized by depression severity. The table has 6 columns: Depression severity, Age, Female, BMI, Total n-3, ALA, EPA, DHA, and n-3 supplement use. Row 1: Depression severity (None (0-4)), n (2438), Age (Mean 55.8, sd 16.4), Female (n 1263, 51.8 percent), BMI (Mean 29.2, sd 6.7), Total n-3 (Mean 2.09, sd 1.34), ALA (Mean 1.90, sd 1.22), EPA (Mean 0.061, sd 0.172), DHA (Mean 0.085, sd 0.234), n-3 supplement use (n 375, 15.4 percent). Row 2: Depression severity (Mild (5-9)), n (739), Age (Mean 50.2, sd 18.2), Female (n 454, 61.4 percent), BMI (Mean 30.6, sd 8.0), Total n-3 (Mean 1.95, sd 1.39), ALA (Mean 1.80, sd 1.32), EPA (Mean 0.050, sd 0.173), DHA (Mean 0.066, sd 0.232), n-3 supplement use (n 98, 13.3 percent). Row 3: Depression severity (Moderate (10-14)), n (279), Age (Mean 47.6, sd 17.8), Female (n 184, 65.9 percent), BMI (Mean 31.5, sd 8.8), Total n-3 (Mean 1.80, sd 1.13), ALA (Mean 1.69, sd 1.04), EPA (Mean 0.035, sd 0.109), DHA (Mean 0.049, sd 0.158), n-3 supplement use (n 27, 9.7 percent). Row 4: Depression severity (Moderately severe (15-19)), n (101), Age (Mean 50.1, sd 17.9), Female (n 64, 63.4 percent), BMI (Mean 32.6, sd 9.2), Total n-3 (Mean 1.85, sd 1.29), ALA (Mean 1.79, sd 1.39), EPA (Mean 0.017, sd 0.049), DHA (Mean 0.020, sd 0.069), n-3 supplement use (n 10, 9.9 percent). Row 5: Depression severity (Severe (20-27)), n (51), Age (Mean 45.1, sd 16.7), Female (n 29, 56.9 percent), BMI (Mean 30.4, sd 8.4), Total n-3 (Mean 1.59, sd 1.26), ALA (Mean 1.50, sd 1.22), EPA (Mean 0.029, sd 0.137), DHA (Mean 0.036, sd 0.184), n-3 supplement use (n 5, 9.8 percent).

Navigate back to Table 1.

## Table 2 Long description

The table presents the results of survey-weighted ordinal logistic regression models examining the association between total n-3 intake and depression severity. It has three rows and four columns. The columns are labeled Model, Adjustment, OR per 1 sd increase, 95% CI, and P. The rows are labeled Model 1, Model 2, and Model 3. Model 1 is unadjusted and not reported due to convergence issues. Model 2 is adjusted for age, sex, and race/ethnicity and also not reported due to convergence issues. Model 3 is fully adjusted and includes age, sex, race/ethnicity, income:poverty ratio, BMI, smoking status, alcohol use, physical activity, and n-3 supplement use. In Model 3, higher total n-3 intake is inversely associated with depression severity, with an OR of 0.865 per 1 sd increase, a 95% CI of 0.761 to 0.983, and a P value of 0.026.

Navigate back to Table 2.

## Table 3 Long description

The table presents the results for individual n-3 fatty acids in fully adjusted models. It has four rows and five columns. The columns are labeled Fatty acid, Unit, OR, 95 % CI, and P. The row labels are ALA (18:3n-3), EPA (20:5n-3), DPA (22:5n-3), and DHA (22:6n-3). Row 1: Fatty acid, ALA; Unit, Per 1 sd increase; OR, 0.918; 95 % CI, 0.767, 1.098; P, 0.348. Row 2: Fatty acid, EPA; Unit, Per 1 sd increase; OR, 0.907; 95 % CI, 0.824, 0.998; P, 0.045. Row 3: Fatty acid, DPA; Unit, Per 1 sd increase; OR, 0.911; 95 % CI, 0.819, 1.012; P, 0.083. Row 4: Fatty acid, DHA; Unit, Per 1 sd increase; OR, 0.919; 95 % CI, 0.841, 1.004; P, 0.061.

Navigate back to Table 3.

## Table 4 Long description

The table presents sensitivity analysis results for the association of total n-3 intake with depression severity. It has three rows and five columns. The columns are labeled Analysis, n, OR per 1 SD increase, 95 % CI, and P. The rows are labeled Main analysis (reference), Excluding n-3 supplement users, and Two dietary recalls only. The values in the table are as follows: Row 1: Main analysis (reference), 3608, 0.865, 0.761, 0.983, 0.026. Row 2: Excluding n-3 supplement users, 3093, 0.872, 0.773, 0.984, 0.026. Row 3: Two dietary recalls only, 3229, 0.847, 0.751, 0.955, 0.007.

Navigate back to Table 4.

## References

[1] World Health Organization (2017) Depression Other Common Mental Disorders: Global Health Estimates. Geneva: WHO.

[2] Hasin DS , Sarvet AL , Meyers JL , et al. (2018) Epidemiology of adult DSM-5 major depressive disorder and its specifiers in the United States. JAMA Psychiatry 75, 336. 29450462 10.1001/jamapsychiatry.2017.4602 PMC5875313

[3] Rush AJ , Trivedi MH , Wisniewski SR , et al. (2006) Acute and longer-term outcomes in depressed outpatients requiring one or several treatment steps: a STAR*D report. Am J Psychiatry 163, 1905–1917. 17074942 10.1176/ajp.2006.163.11.1905

[4] Lai JS , Hiles S , Bisquera A , et al. (2014) A systematic review and meta-analysis of dietary patterns and depression in community-dwelling adults. Am J Clin Nutr 99, 181–197. 24196402 10.3945/ajcn.113.069880

[5] Bayes J , Schloss J & Sibbritt D (2020) Effects of polyphenols in a Mediterranean diet on symptoms of depression: a systematic literature review. Adv Nutr 11, 602–615. 31687743 10.1093/advances/nmz117 PMC7231605

[6] Grosso G , Micek A , Marventano S , et al. (2016) Dietary *n*-3 PUFA, fish consumption and depression: a systematic review and meta-analysis of observational studies. J Affect Disord 205, 269–281. 27544316 10.1016/j.jad.2016.08.011

[7] Liao Y , Xie B , Zhang H , et al. (2019) Efficacy of *n*-3 PUFAs in depression: a meta-analysis. Transl Psychiatry 9, 190. 31383846 10.1038/s41398-019-0515-5 PMC6683166

[8] Bozzatello P , Brignolo E , De Grandi E , et al. (2016) Supplementation with *n*-3 fatty acids in psychiatric disorders: a review of literature data. J Clin Med 5, 67. 27472373 10.3390/jcm5080067 PMC4999787

[9] Parker G , Gibson NA , Brotchie H , et al. (2006) Meta-analysis effects eicosapentaenoic acid (EPA) clinical trials depression. J Clin Psychiatry 163, 969–978.

[10] Sublette ME , Ellis SP , Geant AL , et al. (2011) Meta-analysis of the effects of eicosapentaenoic acid (EPA) in clinical trials in depression. J Clin Psychiatry 72, 1577–1584. 21939614 10.4088/JCP.10m06634 PMC3534764

[11] Kroenke K , Spitzer RL & Williams JB (2001) The PHQ-9: validity of a brief depression severity measure. J Gen Intern Med 16, 606–613. 11556941 10.1046/j.1525-1497.2001.016009606.x PMC1495268

[12] Colangelo LA , He K , Whooley MA , et al. (2009) Higher dietary intake of long-chain *n*-3 polyunsaturated fatty acids is inversely associated with depressive symptoms in women. Nutrition 25, 1011–1019. 19195841 10.1016/j.nut.2008.12.008 PMC2798585

[13] National Academies of Sciences, Engineering, Medicine, Health, Medicine Division, Food, Nutrition Board, et al. (2024) *NHANES Data Analysis Methodology. The Role of Seafood Consumption in Child Growth and Development*. Washington, DC: National Academies Press (US). 39078928

[14] Grajek M , Krupa-Kotara K , Białek-Dratwa A , et al. (2022) Nutrition and mental health: a review of current knowledge about the impact of diet on mental health. Front Nutr 9, 943998. 36071944 10.3389/fnut.2022.943998 PMC9441951

[15] Smorenburg JN , Hodun K , McTavish PV , et al. (2025) EPA/DHA but not ALA reduces visceral adiposity and adipocyte size in high fat diet-induced obese delta-6 desaturase knockout mice. Mol Nutr Food Res 69, e202400721. 39707641 10.1002/mnfr.202400721 PMC11744037

[16] Suominen-Taipale AL , Turunen AW , Partonen T , et al. (2010) Fish consumption and polyunsaturated fatty acids in relation to psychological distress. Int J Epidemiol 39, 494–503. 20156998 10.1093/ije/dyp386 PMC2846446

[17] Kipnis V , Subar AF , Midthune D , et al. (2003) Structure of dietary measurement error: results of the OPEN biomarker study. Am J Epidemiol 158, 14–21; discussion 22–26. 12835281 10.1093/aje/kwg091

[18] Burdge GC & Calder PC (2005) Conversion of *α*-linolenic acid to longer-chain polyunsaturated fatty acids in human adults. Reprod Nutr Dev 45, 581–597. 16188209 10.1051/rnd:2005047

[19] Brenna JT , Salem N Jr , Sinclair AJ , et al. (2009) Alpha-Linolenic acid supplementation and conversion to *n*-3 long-chain polyunsaturated fatty acids in humans. Prostaglandins Leukot Essent Fatty Acids 80, 85–91. 19269799 10.1016/j.plefa.2009.01.004

[20] Mozaffarian D & Rimm EB (2006) Fish intake, contaminants, and human health: evaluating the risks and the benefits. JAMA 296, 1885–1899. 17047219 10.1001/jama.296.15.1885

[21] Peng Z , Zhang C , Yan L , et al. (2020) EPA is more effective than DHA to improve depression-like behavior, Glia cell dysfunction and hippcampal apoptosis signaling in a chronic stress-induced rat model of depression. Int J Mol Sci 21, 1769. 32150824 10.3390/ijms21051769 PMC7084382

[22] Bigornia SJ , Harris WS , Falcón LM , et al. (2016) The *n*-3 index is inversely associated with depressive symptoms among individuals with elevated oxidative stress biomarkers. J Nutr 146, 758–766. 26936135 10.3945/jn.115.222562 PMC4807643

[23] Okereke OI , Vyas CM , Mischoulon D , et al. (2021) Effect of long-term supplementation with marine *n*-3 fatty acids *v.* placebo on risk of depression or clinically relevant depressive symptoms and on change in mood scores: a randomized clinical trial. JAMA 326, 2385–2394. 34932079 10.1001/jama.2021.21187 PMC8693224

[24] Kelaiditis CF , Gibson EL & Dyall SC (2023) Effects of long-chain *n*-3 polyunsaturated fatty acids on reducing anxiety and/or depression in adults; a systematic review and meta-analysis of randomised controlled trials. Prostaglandins Leukot Essent Fatty Acids 192, 102572. 37028202 10.1016/j.plefa.2023.102572

[25] Grosso G , Galvano F , Marventano S , et al. (2014) *n*-3 fatty acids and depression: scientific evidence and biological mechanisms. Oxid Med Cell Longev 2014, 313570. 24757497 10.1155/2014/313570 PMC3976923

[26] Carney RM , Steinmeyer BC , Freedland KE , et al. (2016) Baseline blood levels of *n*-3 and depression remission: a secondary analysis of data from a placebo-controlled trial of *n*-3 supplements: a secondary analysis of data from a placebo-controlled trial of *n*-3 supplements. J Clin Psychiatry 77, e138–e143. 26930527 10.4088/JCP.14m09660 PMC5369023

[27] Reigada LC , Buchanan EM , Hazeltine DB , et al. (2021) A pilot randomized controlled trial testing supplements of *n*-3 fatty acids, probiotics, combination or placebo on symptoms of depression, anxiety and stress. J Affect Disord Rep 5, 100141.

[28] Deane KHO , Jimoh OF , Biswas P , et al. (2021) *n*-3 and polyunsaturated fat for prevention of depression and anxiety symptoms: systematic review and meta-analysis of randomised trials. Br J Psychiatry 218, 135–142. 31647041 10.1192/bjp.2019.234

